# Evaluation of Medication Error Incident Reports at a Tertiary Care Hospital

**DOI:** 10.3390/pharmacy8020069

**Published:** 2020-04-19

**Authors:** Mohammed Aseeri, Ghadeer Banasser, Omar Baduhduh, Sabirin Baksh, Nasser Ghalibi

**Affiliations:** 1 College of Medicine, King Saud bin Abdul Aziz University for Health Sciences, Jeddah 21423, Saudi Arabia; 2 Pharmaceutical Care Services, King Abdul Aziz Medical City, Jeddah 21423, Saudi Arabia; BANASSERGH@ngha.med.sa; 3 Saudi Medication Safety Center, Ministry of National Guard Health Affairs, Riyadh 11426, Saudi Arabia; 4 College of Pharmacy, Ibn Sina National College, Jeddah 22421, Saudi Arabia; omarbaduhduh@gmail.com (O.B.); Sabreenbakhsh36@gmail.com (S.B.); 5 College of Pharmacy, Jazan University, Jazan 45142, Saudi Arabia; Nasserghalibi@gmail.com

**Keywords:** medication error, incident reports, pharmacist

## Abstract

**Background**: Medications errors (MEs) have been a major concern of healthcare systems worldwide. Voluntary-based incident reporting is the mainstay system to detect such events in many institutions. However, the number of reports can be highly variable across institutions depending on their adoption of the safety culture. This study aimed to evaluate and analyze medication error incidents that were submitted through the hospital safety reporting system in 2015 at a tertiary care center in the western region of Saudi Arabia, and to explore the most common types of harmful MEs in addition to the risk factors that led to such harmful incidents. **Methods**: This is a descriptive study that was conducted utilizing 624 medication error reports extracted from the hospital safety reporting system. Reports were analyzed based on the medication name, event type, event description, nodes of the medication use process, harm score (adapted from the National Coordinating Council for Medication Error Reporting and Prevention harm index), patients’ age/gender, incident setting, and time of occurrence as documented in the Safety Reporting System (SRS). Furthermore, all errors that resulted in injury or harm to patients had a deeper review by two senior pharmacists to find contributing factors that led to these harmful incidents and recommend system-based preventive strategies. **Results**: This study showed that most reported incidents were near misses (69.3%). The pediatric population was involved in 28.4% of the incident reports. Most of the reported incidents were categorized as occurring in the inpatient setting (57.4%). Medication error incidents were more likely to be reported in the morning shift versus evening and night shift (77.4% vs. 22.6%). Most reported incidents involved the dispensing stage (36.7%). High-alert medications were reported in 281 out of 624 events (45%). **Conclusions**: The hospital medication safety reporting program is a great tool to identify system-based issues in the medication management system. This study identified many opportunities for improvement in the medication use system, especially in management of chemotherapy and anticoagulant agents.

## 1. Introduction

Medication errors (MEs) have been a major concern of healthcare systems worldwide [1]. It is estimated that serious MEs kill 7000 patients, injure at least 1.5 million, and cost 3.5 billion dollars annually in American hospitals [2]. Compromised patient wellbeing caused by MEs and increased length of hospital stay are factors that led to a profound psychological and financial burden to the patients’ families and healthcare systems [2,3]. According to The National Coordinating Council for Medication Error Reporting and Prevention (NCC MERP) Medication error (ME) is defined as “any preventable event that may cause or lead to inappropriate medication use or patient harm while the medication is in the control of the healthcare professional, patient or consumer. Such events may be related to professional practice, healthcare products, procedures, and systems, including prescribing, order communication, product labeling, packaging, and nomenclature, compounding, dispensing, distribution, administration, education, monitoring, and use” [4]. Medication error reporting, if properly utilized, is a powerful tool for developing and maintaining awareness of MEs risk in healthcare practice. Error reporting helps to unfold the healthcare system’s underlying risks and causes of near-miss or error events [5]. Incident reporting is defined as the action of voluntary reporting upon encountering any event that contradicts the routine or the medication use policies of institutions that may or did result in harm to patient or healthcare provider [6]. Incident reporting is also defined as “a process used to document occurrences that are not consistent with routine hospital operation or patient care”. The process of reporting is represented by filling out an ME report form, which consists of demographic data of the patient, medication involved in the error, and factual description of the medication errors. Since entering an incident report is usually voluntary-based, the number of reports will be different across institutions depending on the safety culture being adopted and does not usually offer an accurate measure of safety performance [5]. For this and several other reasons, the National Coordinating Council for Medication Error Reporting and Prevention (NCC MERP) and the Institute for Safe Medication Practices (ISMP) have discouraged using error rates and benchmarking in medication safety [7,8].

Developing a well-structured internal reporting program is essential. However, devoting resources to analyze and learn from collected data is decisive. During the early 1990s, the concept of system-based causes of medication errors started to become acceptable in healthcare practice [9]. The ISMP has identified the ten Key Elements of the Medication Use System™, which help reveal those system-based causes [10]. These key elements include the following: patient demographic and clinical information like name, age, allergy, and lab results; drug information that should be readily accessible, accurate, and up-to-date; communication of drug information between healthcare providers; drug nomenclature, labeling, and packaging like drug names that sound-alike or look-alike; drug storage, stock, standardization, and distribution; drug device acquisition, use, and monitoring; environmental factors and staffing patterns; staff competency and education; patient education; and, lastly, quality processes and risk management [10]. Applying this concept in analyzing reported medication error events draws attention to system issues rather than blaming individuals and overlooking crucial system pitfalls.

Two locally conducted studies were able to describe the major categories of reported safety incidents [2,6]. The first study assessed overall incident reports, including medication error reports. The most common reported incidents by type were procedural variances (36.8%), behavior and communication incidents (34.3%), hazardous and safety incidents (9.5%), then followed by medication errors (7.4%) [6]. Other studies have aimed to assess the rate of medications errors over a one-year period and resulted in a finding of 0.4% (949 MEs in 240,000 prescriptions) [2]. From all MEs, a percentage of (1.5%) were categorized as harmful errors based on the National Coordinating Council for Medication Error Reporting and Prevention (NCC MERP) index categorization of error outcome. The most common stage in which MEs take place is prescribing and improper dose. Shortage on staffing and heavy workloads were the main contributing factors led to MEs. Finally, the most common types of medications associated with errors were antibiotics, antihypertensive agents, and oral hypoglycemic agents [2].

The aim of this study is to evaluate and analyze medication error incidents that were submitted through the hospital safety reporting system in 2015 at a tertiary care center in the western region of Saudi Arabia, and to explore the most common types of harmful MEs in addition to the risk factors that led to such harmful incidents.

## 2. Materials and Methods

This is a retrospective study evaluating all reported ME incidents over the period of January 2015 to December 2015. Reports were extracted from the hospital Safety Reporting System (SRS) at 700-bed tertiary care hospital in Saudi Arabia. The hospital is a major institution in the western region of Saudi Arabia and have wide varieties of clinical services, including medical, surgical, oncology, emergency medicine, critical care, and pediatrics. The SRS is utilized as a voluntary web-based forms used by healthcare professionals to report many types of safety incidents including MEs. The following data were extracted for each report: event description, stage of error, medication involved, timing of error (i.e., morning, evening, or night shift), care setting (i.e., outpatient or inpatient), action taken by medication safety program quality management, and reported outcomes of MEs. Data collected were analyzed using the SPSS, version 20 (IBM, Armonk, NY, USA). Frequencies, percentages, and association between work shift with both stage of medication use and error outcomes were examined. The National Coordinating Council for Medication Error Reporting and Prevention (NCC MERP) index was used to categorize outcome of medication error [11]. The ISMP list of high alert medications was used to identify medications that bear a heightened risk of causing significant patient harm when used in error [12]. ISMP ten Key Elements of the Medication Use System™ were utilized to identify contributing factors that led to MEs [10]. All MEs that resulted in harm were analyzed by two pharmacists to identify contributing factors that led to these harmful incidents and recommend system-based solutions.

## 3. Results

During the study period, a total of 624 medication error reports were submitted. Medication incidents were reported consistently throughout the year; quarterly total numbers of reports were 21.5%, 28.9%, 20.9%, and 27.4%, for each quarter, respectively. Most of reported incidents were near misses (69.3%), which were intercepted before reaching the patients (Table 1). Staff working in the inpatient setting reported medication errors more than outpatient setting (63.1%; Table 2). Most reported errors were during the morning shift 77.4%, followed by night and evening shifts (17.9% and 6.4%, respectively) (Table 3). High-alert medications were involved in almost half of the reported incidents (45%, n = 281). The most common high alert medication classes involved in incidents reports during the study period were chemotherapeutic agents 23.6%, followed by anticoagulants, and opiates and narcotics (7.5% and 4.8%, respectively) (Table 4). Medication errors occurred at every step of the medication-use processes. Dispensing was reported as most frequently involved stage in medication error (36.7%), followed by prescribing/ordering (34.1%) (Table 5). The most commonly reported event types were delay in medication dispensing/administration (15%), followed by incorrect dose, wrong patient, and dose omission (13%, 8.9%, and 7.5%, respectively) (Table 6).

Reported prescribing errors were significantly associated with daytime, on the other hand, administration reported errors were significantly associated with after-hours (*p* < 0.001) (Table 7). Similarly, there was no apparent trend with reported error outcomes and events time, yet they were all statistically significant (Table 8).

More than half of incidents with harm were reported as dose incorrect (61.5%, n = 8). Identified system underlying causes of harmful medication error included the following: critical drug information missing; critical patient information missing or wrong; drug delivery device problem; drug labeling, naming, or packaging problem; drug storage; lack of quality control or independent check systems; lack of staff competency and education; miscommunication of drug order; and patient education problems. Following is an example of reported medication error with harm.

A pediatric patient was prescribed baclofen 2.5 mg orally twice daily but was filled as 2.5 mL orally twice daily. A baclofen suspension (5 mg/mL) was dispensed and labeled with the following instruction: 2.5 mL orally twice daily. Patient received 12.5 mg (5-fold higher than prescribed dose). Patient took one dose and came to Emergency Department next day very lethargic, was intubated, admitted to Pediatric Intensive Care Unit for 3 days.

Suggested error-reduction strategies included prescribing oral liquid medications using metric weight system only (e.g., mg). Prescribing only by volume (i.e., mL) is ambiguous, and can lead to dosing errors as most medications are available in more than one strength or concentration. This recommendation was built into the Computerized Prescriber Order Entry (CPOE) system.

## 4. Discussion

Proper utilization and analysis of medication error reports provide a valuable insight into system-based pitfalls regardless of the care setting. In our study, the majority of reported medication errors were in the inpatient setting compared to outpatient. This finding might be explained by the variety of unique barriers that hinder medications error detectability in outpatient settings, such as short irregular interactions between patients and healthcare providers and wide diversity of healthcare providers that patients are dealing with in those settings [13]. It was found that hospital staff reported medication errors more in the day shift than after-hours, which could be attributed to the limited number of staff who are assigned to perform specific tasks during after-hours shifts, also clinical services are usually busy during the morning shift than the evening or night shift. The most common stage of medication-use process that was involved in reported events was found to be at the dispensing stage, while 21 studies in the middle east report MEs in the prescribing process to be the highest [14]. This finding may be attributed to the vigilance of our nursing staff who were detecting and reporting dispensing errors before the drug administration. This study showed that nearly half of the reported errors involved high alert medications. Anticoagulant agents were one of the most common types of medication involved in ME reports, similar to the finding by Khalili et al. [15]. There was a statistically significant relationship between the higher reported near-miss incidents in the morning shift than after-hours. However, harmful incident reports were more in after-hours than in the daytime. The later result was found to be statistically significant, as well. Miscommunication, missing critical drug/patient information, and lack of independent double check at the time of administration were significant contributing factors led to a harmful incident in this study.

## 5. Conclusions

MEs are one of the massive risks facing patient safety around the world. Preventing them may be almost impossible but reducing them should be a priority for directors of healthcare organizations. The hospital medication safety reporting program is a great tool to identify system-based issues in the medication management system. However, it requires more effort from the healthcare providers to implement the system. This study identified many opportunities for development in the medication use system, especially in management of chemotherapy and anticoagulant agents.

## 6. Recommendations

1—Hospital administration should make great efforts to promote medication safety culture and encourage hospital staff to report all medication errors whether they are near misses, or actual errors reached the patients.

2—The medication safety program should obtain more information about the incident when it occurred, identify contributing factors, and send feedback to the reported department/staff with system-based solution to avoid these errors from occurring again.

3—Implementing of appreciation award to enhance error reporting in the hospital setting.

4—Contributing factors involved in harmful events need to be discussed in detail in future studies.

## Figures and Tables

**Table 1:** Medication error incident reports classified by degree of patient harm according to National Coordinating Council for Medication Error Reporting and Prevention (NCC MERP).

NCC MERP Category	Definition	Classification	n	%
A	Circumstances or events that have the capacity to cause error (near miss)	No error	202	32.4
B	An error occurred, but the error did not reach the patient (near miss)	Error, no harm	230	36.9
C	An error occurred that reached the patient but did not cause patient harm		76	12.2
D	An error occurred that reached the patient and required monitoring to confirm that it resulted in no harm to the patient and/or required intervention to preclude harm		103	16.5
E	An error occurred that may have contributed to or resulted in temporary harm to the patient and required intervention	Error, harm	6	1
F	An error occurred that may have contributed to or resulted in temporary harm to the patient and required initial or prolonged hospitalization		6	1
G	An error occurred that may have contributed to or resulted in permanent patient harm		1	0.2
H	An error occurred that required intervention necessary to sustain life		0	0
I	An error occurred that may have contributed to or resulted in the patient’s death	Error, death	0	0
Total			624	100

NCC MERP National Coordinating Council for Medication Error Reporting and Prevention.

**Table 2:** Medication errors by care setting.

Setting	n	%
In-patient setting	394	63.1
Out-patient setting	230	36.9
Total	624	100

**Table 3:** Working shifts and medication error incidents reports.

Shit Time	n	%
Morning	483	77.4
Evening	29	6.4
Night	112	17.9
Total	624	100

**Table 4:** High-alert medication/class involved in reported incidents.

Class of High Alert Medication	n	%
Chemotherapeutic agents	147	23.6
Opiates and narcotics	30	4.8
Insulin	16	2.6
Anesthetic agents	4	0.6
Adrenergic antagonists	3	0.5
Electrolytes	24	3.8
Total parenteral nutrition preparations	5	0.8
Anticoagulants	47	7.5
Antiarrhythmics	2	0.3
Adrenergic agonists	1	0.2
Neuromuscular blocking agents	1	0.2
Dextrose	1	0.2
Total	624	100

**Table 5:** Reported stage of medication use processes.

Stage of Error	n	%
Not applicable	3	0.5
Prescribing/ordering	213	34.1
Transcribing	17	2.7
Dispensing	229	36.7
Administering	74	11.9
Monitoring	8	1.3
Other	80	12.8
Total	624	100

**Table 6:** Reported specific event types.

Event Type	n	%
Dose Omission	47	7.53
incorrect dose	82	13.14
Dose-extra/duplication	35	5.61
Dose-incorrect concentration/strength	20	3.21
Medication-incorrect	48	7.69
Dosage form- incorrect	8	1.28
Other	75	12.02
Delay	96	15.38
Duration-incorrect	11	1.76
Wrong Patient	56	8.97
Authorization problem	12	1.92
Deteriorated Drug- damaged	5	0.8
Allergy-known-patient ordered/received med	2	0.32
Drug out of stock	18	2.88
Medication-expired/outdated	10	1.6
Medication-incompatible	2	0.32
Medication-package issue	37	5.93
incorrect rout of administration	4	0.64
Allergy not documented	6	0.96
Frequency-incorrect	9	1.44
Medication-Given without order	3	0.48
Medication-discontinued	4	0.64
Medication lost	4	0.64
Contraindicated-drug-disease interactions	2	0.32
Incomplete Order	2	0.32
Medication-duplicate therapeutic category	8	1.28
Labeling issue	18	2.88
Total	624	100

**Table 7:** Association between the incident shift and stage of medication use.

Stage of Error	Daytime	After-Hours	*p* Value
Prescribing	38.70%	18.40%	*p* < 0.001
Dispensing	32.90%	49.60%	
Administering	9.70%	19.10%	

**Table 8:** Association between the incident shift and error outcome.

Error Outcome	Daytime	After-Hours	*p* Value
Near miss	71.30%	59.60%	*p* = 0.004
Error with no harm	25.90%	36.90%	
Error with harm	2%	3.50%	
